# "SARCOPENIA MEASURED BY ULTRASOUND IN HOSPITALIZED OLDER ADULTS" (ECOSARC): multi-centre, prospective observational study protocol

**DOI:** 10.1186/s12877-023-03891-5

**Published:** 2023-03-22

**Authors:** Esther López Jiménez, Marta Neira Álvarez, Raquel Ramírez Martín, Cristina Alonso Bouzón, María Solange Amor Andrés, Cristina Bermejo Boixareu, Fátima Brañas, Rocío Menéndez Colino, Estefanía Arias Muñana, Marta Checa López, Concha Grau Jiménez, Patricia Pérez Rodríguez, María Alcantud Ibáñez, Brian Vasquez Brolen, Juan Oliva, Luz María Peña Longobardo, Rubén Alcantud Córcoles, Elisa Belén Cortés Zamora, Elena Gómez Jiménez, Luis Romero Rizos, Almudena Avendaño Céspedes, Carmen Rosa Hernández Socorro, Pedro Abizanda

**Affiliations:** 1 Complejo Hospitalario Universitario de Albacete, C/ Seminario 4, 02006 Albacete, Spain; 2 Hospital Universitario Infanta Sofía, San Sebastián de los Reyes, Madrid, Spain; 3 Hospital Universitario La Paz, Madrid, Spain; 4 Hospital Universitario de Getafe, Getafe, Madrid, Spain; 5 Hospital Virgen del Valle, Toledo, Spain; 6 Hospital Universitario Puerta de Hierro, Majadahonda, Madrid, Spain; 7 Hospital Universitario Infanta Leonor, Madrid, Spain; 8 Department of Economic Analysis and Finance, Universidad de Castilla-La Mancha, Toledo, Spain; 9 Centro de Investigación Biomédica en Red de Fragilidad y Envejecimiento Saludable (CIBERFES), Instituto de Salud Carlos III, Madrid, Spain; 10 Fundación Hospital Nacional de Parapléjicos, Toledo, Spain; 11 Facultad de Medicina de Albacete, Universidad de Castilla-La Mancha, Albacete, Spain; 12 Facultad de Enfermería de Albacete, Universidad de Castilla-La Mancha, Albacete, Spain; 13 Hospital Universitario de Gran Canaria, Doctor Negrín, Las Palmas de Gran Canaria, Spain

**Keywords:** Sarcopenia, Ultrasound, Echography, Hospitalization, Older adults, Quadriceps rectus femoris, Outcomes, Prospective

## Abstract

**Background:**

Measurement of muscle mass and function, and thereafter, screening and diagnosis of sarcopenia, is a challenge and a need in hospitalized older adults. However, it is difficult in complex real-world old patients, because usually they are unable to collaborate with clinical, functional, and imaging testing. Ultrasound measurement of *quadriceps rectus femoris* (QRF) provides a non-invasive, real-time assessment of muscle quantity and quality, and is highly acceptable to participants with excellent inter-rater and intra-rater variability. However, normative data, protocol standardization, and association with longitudinal outcomes, needs further research and consensus.

**Methods:**

Prospective exploratory multicenter study in older adults admitted to Acute Geriatric Units (AGUs) for medical reasons. 157 subjects from 7 AGUs of Spain were recruited between May 2019 and January 2022. Muscle ultrasound measurements of the anterior vastus of the QRF were acquired on admission and on discharge, using a previously validated protocol, using a Chieson model ECO2 ultrasound system (Chieson Medical Technologies, Co. Ltd, Wimxu District Wuxi, Jiangsu, China). Measurements included the cross-sectional area, muscle thickness in longitudinal view, intramuscular central tendon thickness, echogenicity, and the presence or absence of edema and fasciculations. Functional, nutritional, and DXA measurements were provided. Clinical follow-up was completed at discharge, and 30 and 90 days after discharge. Variations between hospital admission and discharge ultrasound values, and the relationship with clinical variables, will be analyzed using paired *t*-tests, Wilcoxon tests, or Mc Nemar chi-square tests when necessary. Prevalence of sarcopenia will be calculated, as well as sensitivity and specificity of ultrasound measurements to determine sarcopenia. Kappa analysis will be used to analyze the concordance between measurements, and sensitivity analysis will be conducted for each participating center.

**Discussion:**

The results obtained will be of great interest to the scientific geriatric community to assess the utility and validity of ultrasound measurements for the detection and follow-up of sarcopenia in hospitalized older adults, and its association with adverse outcomes.

**Trial registration:**

NCT05113758. Registration date: November 9^th^ 2021. Retrospectively registered.

## Background

The human aging process involves a decrease in lean mass together with a parallel increase in fat mass [1]. The skeletal muscle system from the third decade of life onwards, undergoes a slow but progressive loss of muscle mass and strength, a circumstance that is accentuated from the age of 65–70 years [2]. From the age of 50, muscle mass decreases by 1–2% annually, and muscle strength by 1.5–3% [3]. In men the process is more progressive, while in women there is an abrupt decrease coinciding with the menopause [3]. This process of loss of muscle mass was defined as sarcopenia [4], and since 2016 it is recognized as an independent condition by an International Classification of Disease, Tenth Revision, Clinical Modification (ICD-10-CM) Code (code M62.84) [5].

Although a consensus global definition of sarcopenia is still evolving [6], the European Working Group on Sarcopenia in Older People (EWGSOP) considers it as a “muscle disease (muscle failure) rooted in adverse muscle changes that accrue across a lifetime. Low muscle strength is the key characteristic of sarcopenia, the detection of low muscle quantity and quality in used to confirm the sarcopenia diagnosis, and poor physical performance is indicative of severe sarcopenia” [7]. This lack of consensus must not hinder the importance of diagnosing and treating sarcopenia in clinical practice [6, 8], because of its demonstrated association with falls and fractures, disability in activities of daily living (ADL), frailty, mobility disorders, cardiac and respiratory diseases, cognitive impairment, institutionalization, quality of life, healthcare costs, and death [7, 9–12].

Several tools are being used for the assessment of muscle mass and quality, including bioelectrical impedance analysis (BIA), magnetic resonance imaging (MRI), computed tomography (CT), dual energy x-ray absorptiometry (DXA), and ultrasound imaging. Recent publications present that ultrasonography may be a valid and reliable imaging method for the assessment of skeletal muscle mass [13–15].

However, the emerging field of ultrasound assessment of muscle mass and quality makes mandatory the need for a standardization of the measurement technique, the identification of the best muscles for determinations, and the cut-off values in different populations, settings, and conditions [16]. Ultrasonography is an easy portable technique, non-invasive, radiation-free, and cost-effective, that not only measures thickness, cross-sectional area and volume, fascicle length, and pennation angle, but also can determine mechanical properties, echogenicity (fat infiltration, fibrosis, myonecrosis), and microcirculation [13]. In older adults, changes in all these parameters can be detected compared to younger adults, mainly in lower limb muscles with antigravitary function, such as the quadriceps femoris and gastrocnemius medialis. Thereafter, these parameters could be theoretically useful for detecting sarcopenia in this population [17].

However, regarding muscle ultrasound measurements in older populations, many questions remain active. The prevalence of sarcopenia ranges from 10 to 40%, with wide ranges due to different measurement methodologies, diagnostic criteria, settings, ethnicity, age, sex, clinical conditions, and selected populations [14, 18]. Reliability and validity values from ultrasound studies have been obtained under strictly controlled conditions, which are likely to decrease in real clinical practice [13, 19], although ultrasound feasibility in acute geriatric settings has been analyzed with promising results [20]. Acknowledging that muscle quality measurements (ability of the tissue to perform its functions including contraction, metabolism, and electrical conduction) seem more important than quantity parameters, functional determinations remain a challenge [21].

Hospitalization for acute illnesses significantly affect the muscle status of critically ill patients [22], and the occurrence of incident sarcopenia in older adults measured with BIA [23], but longitudinal information with ultrasonography in hospitalized older adults is lacking. Finally, imaging diagnostic techniques like DXA, BIA, CT or MRI, may be difficult to acquire in hospitalized older adults, while new assessment protocols using ultrasound imaging have been described in Critical Care Units [24].

Consequently, the aim of the ECOSARC Project is to longitudinally estimate, by means of portable ultrasound under real-life conditions, parameters of muscle quantity and quality (rectus femoris muscle area, thickness and echostructure) in older adults hospitalized in Acute Geriatric Units (AGUs) for medical reasons, the muscle changes between admission and discharge, the prevalence of sarcopenia on admission, the association between muscle parameters with clinical variables and outcomes, and the correlation between ultrasound measurements and other techniques like DXA.

## Methods/design

### Objectives

The main objective of the study is to estimate, by means of portable ultrasound, parameters of muscle quantity and quality of the anterior vastus of the quadriceps rectus femoris (avQRF), including muscle area, thickness, and echostructure, in older adults hospitalized in AGUs for medical reasons.

Secondary objectives are 1) to determine the prevalence of sarcopenia on admission, using the EWGSOP-2 criteria [7], 2) to determine the incidence of sarcopenia on discharge, 3) to assess the evolution of muscle mass of the avQRF between admission and discharge, 4) to evaluate changes in nutritional status, lab results, hand grip strength, gait speed, ADL, frailty, and physical performance from admission to discharge, and at 30 and 90 days for ADL and frailty, and their relationship with ultrasound muscle measurements, and 5) to analyze the relationship between ultrasound muscle measurements and other methods of muscle assessment like DXA or SARC-F. These analyses will help in the validation process of ultrasonography for muscle mass and quality assessment.

### Study design

Prospective exploratory multicenter study in older adults admitted to an AGU.

### Setting

The study was conducted in the Geriatric Departments of 7 hospitals in Spain: Complejo Hospitalario Universitario de Albacete (Albacete), Hospital Universitario de Getafe (Getafe), Hospital Universitario Infanta Sofía (San Sebastián de los Reyes), Hospital Universitario Infanta Leonor (Madrid), Hospital Universitario La Paz (Madrid), Hospital Universitario Puerta de Hierro (Majadahonda), and Hospital Virgen del Valle (Toledo).

### Participants

Patients admitted to an AGU of the 7 participating centers between May 2019 and January 2022, meeting the inclusion criteria: (i) Age ≥ 69 years, (ii) Holden's Functional Ambulation Classification Scale level 1 or higher, and (iii) Informed consent signed by the patient or legal representative.

Exclusion criteria were: (i) Terminal condition or life expectancy of less than 6 months, (ii) impossibility or refusal to undergo muscle ultrasound, (iii) refusal for follow-up, (iv) severe dementia stage, with a Reisberg´s Global Deterioration Scale (GDS) scoring 7, and/or (v) impossibility in the opinion of the investigators to complete the necessary procedures of the study.

### Measurements

Baseline and discharge data were collected from patients and caregivers in person by a research Geriatrician and from the patients’ clinical records. Baseline patient characteristics were evaluated 15 days pre-hospitalization by asking retrospectively the patient or their caregivers/families/legal representatives when necessary. Follow-up data were collected by the researchers by telephone at 30 and 90 days after discharge in order to know their health status (Table 1).

**Table 1:** Scheduled visits and activities

	**15 days pre-hospitalization**	**Baseline**	**Discharge**	**30 days post-discharge**	**90 days post-discharge**
Sociodemographic data		X			
Clinical data on admission		X			
Clinical data at discharge			X		
Conditions during admission			X		
Follow-up outcomes (mortality, number of visits to Primary Care physician or Nurse, Emergency Department visits, falls, and hospital readmissions)				X	X
Holden Functional Ambulation Classification (FAC)		X			
Charlson Index		X			
Barthel Index	X	X	X	X	X
Reisberg Global Deterioration Scale (GDS)	X				
FRAIL instrument	X	X	X	X	X
SARC-F scale	X				
Mini Nutritional Assessment- Short Form (MNA-SF®)		X			
Short Physical Performance Battery (SPPB)		X	X		
Hand grip strength (kg)		X	X		
Laboratory results on admission		X			
Ultrasound measurements		X	X		
DXA data		X	X		
Semi-quantitative intake questionnaire		X	X		

### Independent variables

- Demographic, social, and anthropometric data: date of birth, sex, weight, height, body mass index (BMI), ethnicity, lives alone or accompanied, lives at home or in a long-term care. We will use these variables as control ones.
- Holden Functional Ambulation Classification (FAC) [25] is a functional assessment classification of gait ability, that quantifies the degree of gait dependence from 0 (totally dependent and unable to walk alone) to 5 (totally independent walking). This variable will be used as inclusion criteria.
- Charlson Comorbidity Index: this is a system for evaluating life expectancy at 10 years, depending on the age of the subject being evaluated and the comorbidities present [26]. It consists of 19 items (apart from age), which if present influence the subject's life expectancy. Initially it was adapted to assess survival at 1 year, but later in its final form it was adapted to assess survival at 10 years [27]. Each item has a given score, and the sum establishes the degree of comorbidity. In general, absence of comorbidity is considered to be 0–1 point, low comorbidity, 2 points, and high comorbidity equal to or greater than 3 points. Survival at 10 years is calculated using calculators [28]. This variable will be used in multivariate analysis as a control one.
- Clinical data not included in the Charlson index were recorded, including hip prosthesis, spinal estenosis or myelopathy, osteoporosis (or requires medication), vitamin D deficiency (or requires drug or nutritional supplementation), type of dementia if applicable (Alzheimer's disease, vascular or mixed), dyslipidemia (or drug required), insomnia (or requires medication), depressive syndrome (or requires medication), hearing deficit, complete deafness, significant vision loss, blindness, parkinsonism, epilepsy, and other relevant pathologies. This variable will be used in multivariate analysis as a control one.
- Main clinical diagnosis at the time of hospital admission. This variable will be used in multivariate analysis as a control one.
- Drugs prescribed up to the time of admission to the hospital. This variable will be used in multivariate analysis as a control one.
- Barthel Index: the Barthel index assesses basic ADL [29]. It is a hetero-administered questionnaire with 10 Likert-type items. The range of possible index values is between 0 and 100, with 5-point intervals, being the lower the score, the greater the dependence. This variable will be used to evaluate the secondary objective number 4.
- Reisberg GDS: it is the most widely used instrument to assess cognitive status and detect the presence of dementia of any etiology, although it was initially constructed to assess Alzheimer's disease [30]. It consists of 7 categories ranging from no cognitive impairment (GDS 1) to very severe cognitive impairment (GDS 7), and is evaluated by the physician. This variable will be used as inclusion criteria.
- FRAIL instrument: it is a simple frailty screening questionnaire developed by the Consensus Conference on Frailty working group and consists of 5 simple questions [31]. The answer can only be yes (scores 1 point) or no (0 points). 0 points indicates no frailty, 1–2 points prefrailty, and 3 or more points identify frailty. This variable will be used to evaluate the secondary objective number 4, and also as a control variable in multivariate analysis.
- SARC-F instrument: used for sarcopenia screening, understood as low muscle performance in the presence of a loss of muscle mass. It is a simple 5-question instrument, with three possible answers (score 0, 1 or 2 points) [32]. A score of 4 or higher is considered sarcopenia. This variable will be used to evaluate the secondary objective number 5.
- Short Physical Performance Battery (SPPB): developed by the National Institute on Aging, is an objective assessment tool for evaluating lower extremity physical performance in the older adult [33]. The SPPB consists of three tests: 1) hierarchical balance test, 2) short 4-m walk at usual pace, and 3) rising from a chair five consecutive times. Scores range from 0 (worst performance) to 12 (best performance). Low scores on the SPPB have high predictive value for a wide range of health consequences including disability in ADL, loss of mobility, hospitalization, length of hospitalization, nursing facility admission, and death. This variable will be used to evaluate the secondary objective number 4.
- Hand grip strength (kg) is an indicator of global strength, nutritional status, and predictor of changes in functionality in older adults. A digital JAMAR dynamometer was used. This assessment is performed seated in a chair, with shoulder and forearm in neutral position and elbow in 90 degrees of flexion. The position of the dynamometer was determined according to the size of the hand, allowing a comfortable and functional grip of the instrument with an adequate closure of the metacarpophalangeal and interphalangeal joints in the first position, favoring the contact between the first phalanx of the index finger and thumb. The participant performed a maximum grip force for 3 s, with 1 min rest between each repetition, making two attempts, where the best of both is the one that was used for the study [34]. This variable will be used to evaluate the secondary objective number 4.
- Eating Assessment Tool- 10 questionnaire (EAT-10®): this is a verbal, analog, unidimensional, direct-scored self-assessment scale to evaluate specific symptoms of dysphagia [35]. It is a 10-question questionnaire. The patient must respond to each question on a 5-point scale (0–4 points), with 0 indicating no problem and 4 indicating a serious problem. Higher scores indicate greater perception of dysphagia. This variable will be used to evaluate the secondary objective number 4.
- Mini Nutritional Assessment Tool—Short Form Questionnaire (MNA-SF®): this is a set of questions and measurements that assesses nutritional status in a simple way. It is a validated instrument where the highest score indicates normal nutritional status (12–14 points) and the lowest score indicates malnutrition (0–7 points) [36–38]. This variable will be used to evaluate the secondary objective number 4.
- A Semi-quantitative intake questionnaire was recorded daily in selected centers. This questionnaire determines daily the percentage of prescribed food that has been eaten in each meal of the day with five categories [all (> 80%), almost all (80–60%), half (60–40%), almost nothing (40–20%) or nothing (< 20%)]. It has to be performed by a dietitian, and calculates the mean of the complete hospital stay intake, to yield a final percentage of this intake [39]. This variable will be used to evaluate the secondary objective number 4.
- Lab tests data (in each center, usual admission lab parameters were used): hemoglobin, platelets, leukocytes, neutrophils, lymphocytes, albumin, total protein, total cholesterol (LDL and HDL if available), transferrin, ferritin, erythrocyte sedimentation rate (ESR), C-reactive protein (CRP), glycemia, creatinine, urea, thyroid hormones, creatin-phosphokinase, folic acid, and vitamin B12. These variables will be used to evaluate the secondary objective number 4.
- DXA was realized in selected centers (Horizon® DXA system, Hologic Inc., U.S.): variables included in the analysis were total muscle mass, appendicular lean mass (aLM), aLM/Body Mass Inex (BMI), aLM/height^2^, total fat mass, percentage of fat mass (%), start date, end date. These variables will be used to evaluate the secondary objective number 5.
- Patient conditions during hospital admission: number of days of bed rest, bed-chair, and with mobilization; number of days of rehabilitation, occupational therapy, and physical exercise; number of days fasting, liquid diet and basal diet. These variables will be used as possible explicative control variables for muscle changes during hospitalization.

### Muscle ultrasound measurements

Muscle Ultrasound measurements were acquired using a protocol previously validated [24], using a Chieson model ECO2 ultrasound system (Chieson Medical Technologies, Co. Ltd, Wimxu District Wuxi, Jiangsu, China) and a multifrequency linear-array probe (width of probe 38–58 mm). The patients laid supine in the bed with their arms supinated and knees extended and relaxed to full extension. The probe was coated with an adequate water-soluble transmission gel, and was aligned perpendicularly to the longitudinal and transversal axes of avQRF, with the aim of obtaining transverse and longitudinal images. The assessment was done without compression, because muscle dimensions change with contraction/relaxation, and the studied muscle is more compressible in a relaxed state. The acquisition site was located two-thirds of the way along the femur length, measured between the upper pole of the patella and the anterior superior iliac spine. Several images were registered of both QRF muscles for each measurement site. The image was stored pseudononymised on the ultrasound device computer.

The following measurements were acquired: first, we measured the cross-sectional area of the avQRF (mode b, in cm^2^); second, muscle thickness in longitudinal view (mode b, in cm); third, intramuscular central tendon thickness in mm, with an insonation angle perpendicular to the tendon; fourth, echogenicity of the muscle (1. normal; 2. heterogeneous; 3. fat infiltration; 4. atrophy due to fasciitis and necrosis); fifth, we scanned for the presence or absence of edema in the subcutaneous cellular tissue and the intramuscular and intrafascial fluid; sixth and last, we determined the presence or absence of muscle fasciculations using video testing [24].

New research has provided quantitative values and cut-off points of avQRF in older adults in different settings. In a cross-sectional study in 119 community-dwelling older adults from Siena (Italy), average avQRF thickness was 0.78 ± 0.26, significantly lower in sarcopenic patients (0.55 ± 0.2 vs. 0.9 ± 0.3) and females (0.7 ± 0.3 vs 0.86 ± 0.3), with a cut-off point of 0.7 cm for females and 0.9 cm for males to assess the presence of sarcopenia by ultrasound, sensitivity (S) 100%, and specificity (E) 64% [40]. In 857 community-dwelling adults aged 60 years and older from Thailand who were diagnosed with sarcopenia using the Asian Working Group for Sarcopenia-2019 algorithm, cut-off values of ≤ 1.1 cm of avQRF thickness were used for male and ≤ 1 cm for female (S 90.9%, E 92.2% for sarcopenia diagnosis) [41]. Another study in 204 community-dwelling older adults in Kyoto (Japan), showed mean values of avQRF of 2.91 ± 0.50 cm in men and 2.53 ± 0.44 cm in women. Best cut-off points for low skeletal muscle index were 2.88 cm for men (S 0.92, E 0.57) and 2.34 cm for women (S 0.66, E 0.75) [42]. A study in Sichuan (China) including 103 older adults at risk for sarcopenia according the Asian Working Group for Sarcopenia 2019 guidelines, showed a mean avQRF thickness of 2.97 ± 0.78 cm in men and 2.62 ± 0.71 cm in women [43]. Finally, a recent study in 40 older adults in a post-acute care unit in Barcelona (Spain) described a mean (SD) avQRF thickness of 1.49 (0.34) cm for men, and 1.32 (0.29) cm for women, and a avQRF cross-sectional area of 5.3 (1.3) cm^2^ for men, and 4.7 (1.5) cm^2^ for women [44]. We will compare our results with these previous data.

### Outcome measures

At discharge, mortality, length of hospital stay, and final clinical diagnosis were assessed. In addition, participants were followed-up by telephone 30 and 90 days after discharge, by the Geriatricians, to know their health status. Data were provided by the patients or the caregiver when needed. Vital situation and the following outcomes were recorded: falls (with or without fracture), disability in ADLs using the Barthel index, frailty using the FRAIL instrument, visits to the family physician and to specialists, visits to the emergency room, and hospital readmissions. Information was also compared with the electronic health records of the hospital.

### Bias

There could be a bias in the caption and measurements of the ultrasound images. To avoid these biases, geriatricians collecting the images received training by an expert radiologist before the inclusion of participants. In addition, all images were reviewed by an expert radiologist, who confirmed that the acquisition and measurements were correct.

### Data collection procedure

All patients or legal representatives and informal caregivers reviewed, signed, and dated the most current Institutional Review Board (IRB) approved written informed consent for participation in the study before any study-specific screening tests or evaluations were performed. Informed Consent Forms for enrolled patients and their informal caregivers, and for those who are not subsequently enrolled, will be maintained at the study site in compliance with the local data governance regulations.

All screening evaluations were completed and reviewed to confirm that a patient met all eligibility criteria before inclusion.

### Data collection methods

#### Data collection

Data collection was performed by the principal investigator and sub-investigators at each center. Inclusion and exclusion criteria were reviewed in the electronic clinical records at each center. A patient information sheet and informed consent was given to the patient. An evaluation was performed on admission, retrospectively 15 days prior to hospitalization, at discharge, and at 30 and 90 days after discharge as follow-up. All data were recorded in the patient's medical record and subsequently added to an electronic database. Before the study initiation, training was performed by all the researchers to harmonize the data acquisition. In addition, monitoring was conducted during the study procedures to improve adherence to the methodology.

#### Data management

Clinical data, including images, were pseudononymised and securely stored centrally in the electronic Case Report Form (eCRF) designed for this purpose, into a secure electronic Research Electronic Data Capture (REDCap®) [45]. Iidentifiable personal data were pseudononymized by assigning research numbers to the participants. All the investigators responsible for the study at their hospital sites received a personal username and password for the electronic database to handle the data with confidentiality. Access to the whole database was limited to the principal investigator.

### Data monitoring

The complete research team received training for the collection and storage of study data. The principal investigator, who had full access to the complete study registry, supervised the data continuously. The other investigators only had access to the patient registry of their hospital. The study coordinator, who was delegated by the principal investigator, conducted the monitoring.

### Sample size calculation

The main objective was to estimate quantitative parameters of avQRF, mainly the cross-sectional area values. As there were no previous normative data regarding muscle ultrasound values in hospitalized older patients, and also because our study was exploratory, we used information from previous research in Intensive Care Units to calculate the sample size. In the study from Baston et al., using Bland–Altman plots for inter-rater reliability, a measurement bias of 0.2 cm^2^ for the cross-sectional area of avQRF was described [46], allowing us to use this surrogate measure as the minimum significant value to detect real differences between measurements. Accepting an alpha risk of 0.05 and a beta risk of 0.2 in a two-sided test, 157 subjects were necessary to recognize as statistically significant a difference greater than or equal to 0.2 cm^2^ in the cross-sectional area of the avQRF. The standard deviation was assumed to be 0.8. It was anticipated a drop-out rate of 20%.

### Statistical methods

No value imputation system was established, so the final sample size was the number of patients with values at admission and discharge for the different parameters measured. We will determine the normal distribution goodness-of-fit of the continuous variables using the Kolmogorov–Smirnov and Shapiro–Wilk tests as needed. We will calculate the mean and standard deviations in the case of normally distributed variables and the median and the interquartile range in skewed variables. 95% confidence intervals will be calculated when necessary.

Variations between hospital admission and discharge ultrasound values, DXA and anthropometric parameters, functional scales, nutritional status, cognitive status, and in general any other quantitative variable, will be calculated using paired *t*-tests, Wilcoxon tests, or Mc Nemar chi-square tests when necessary.

The prevalence of sarcopenia will be calculated as percentages. Sensitivity and specificity of ultrasound measurements to determine sarcopenia will be calculated. Kappa analysis will be used to analyze the concordance between measurements. The coefficient of variation of the ultrasound device will be calculated. Sensitivity analysis will be conducted for each participating center. All analyses will be performed with SPSS (Statistical Package for Social Sciences) statistical software, version 24.0 or later (IBM Corp. Released 2016. IBM SPSS Statistics for Windows, Version 24.0. Armonk, NY: IBM Corp).

## Discussion

Measurement of muscle mass and function, and thereafter, identification of sarcopenia, is a challenge in hospitalized older adults [47], and multiple studies have demonstrated that it is a disease with clinical relevance in this population, associated to severe clinical outcomes [7, 9–12]. Sarcopenia is frequent in hospitalized older patients, a vulnerable population to this condition that may be present on admission or developed during hospitalization because of hospital-associated immobility [48].

In addition, sarcopenia research is difficult in complex real-world old patients, because usually they are unable to collaborate with clinical, functional, and imaging testing [47]. Measurement of grip strength, and physical function tests like gait speed, the Timed Get Up and Go test, the 5-sit-to-stand test, or the SPPB, all of them necessary to implement sarcopenia criteria [7], may be very difficult to complete in many inpatients. Moreover, DXA, BIA, CT or MRI measurements may be unaccesible to many of them. The procedure with the overall highest completion rates in a recent cohort study was ultrasound measurement of the quadriceps muscle [47]. This technique provides non-invasive, real-time assessment of muscle quantity and quality, is highly acceptable to participants, and is associated with low perceived burden when compared to other testing [49]. Ultrasound measurement of avQRF has excellent inter-rater and intra-rater variability [50, 51], and has been proposed by EWGSOP-2 for clinical assessment of sarcopenia. Different protocols for ultrasound measurements have been proposed, although final consensus is lacking, while standardization is necessary [16, 24, 51–53].

Ultrasound technology has previously demonstrated validity and reliability for muscle mass and quality assessment [13–16, 19, 20, 54–56]. In our study we used a new simple protocol that has been previously described and validated in young adults in ICU patients [24], able to quantify muscle quantity and quality. In addition, we will be able to provide associations with DXA values and physical function tests. Added value of our study will be the multicenter and longitudinal design, and the ultrasound testing acquisition by previously trained geriatricians instead of radiologists, supporting the real-life use of this technique.

The main limitations of the study is the unknown feasibility of the selected protocol for its use in hospitalized older adults, the possibility of a low inter-rater reliability between geriatricians albeit the training, or the heterogeneity of older adults regarding pathologies, functional status or medicines, because our study is non-interventional, only exploratory. Another limitation is that we will not be able to differentiate between sarcopenia and cachexia, although this is not the objective of the study. Cachexia is a common manifestation of several serious illnesses, such as chronic heart failure or cancer, it is associated with chronic inflammation, and in many cases it is related with hospitalization. However, sarcopenia and cachexia can overlap in the same older patient, and differential diagnosis might be difficult in clinical practice, as there is no clear demarcation line between them, or screening and diagnostic tools to clearly identify them [57, 58].

However, we think that the results obtained will be of great interest to the scientific geriatric community to assess the utility of ultrasound measurements for the detection and follow-up of sarcopenia in hospitalized older adults. Future research will need to validate the measurement methodology, describe cut-off points, and identify future strategies for management.

## Data Availability

Data collected and used for this study can be made available upon reasonable request for validation purposes or non-commercial research, when permitted by the participating centers. Requests have to be sent to the corresponding author: Pedro Abizanda: pabizanda@sescam.jccm.es.

## Abbreviations

ICD-10-CM: International Classification of Disease, Tenth Revision, Clinical Modification
EWGSOP: European Working Group on Sarcopenia in Older People
ADL: Activities of Daily Living
BIA: Bioelectrical Impedance Analysis
MRI: Magnetic Resonance Imaging
CT: Computed Tomography
DXA: Dual energy X-ray Absorptiometry
AGU: Acute Geriatric Units
avQRF: Anterior vastus of the Quadriceps Rectus Femoris
GDS: Global Deterioration Scale from Reisberg
BMI: Body Mass Index
FAC: Holden Functional Ambulation Classification
SPPB: Short Physical Performance Battery (SPPB)
Kg: Kilograms
EAT-10: Eating Assessment Tool- 10 questionnaire
MNA-SF: Mini Nutritional Assessment Tool – Short Form
LDL: Low-Density Lipoproteins
HDL: High-Density Lipoproteins
ESR: Erythrocyte sedimentation rate
CRP: C-reactive protein
aLM: Appendicular lean mass
mm: Millimeters
QRF: Quadriceps rectus femoris
cm: Centimeters
BMI: Body mass index
S: Sensitivity
E: Specifity
IRB: Institutional Review Board
eCRF: Electronic Case Report Form
REDCap: Research Electronic Data Capture
SPSS: Statistical Package for Social Sciences
